# 

**Published:** 1906-05

**Authors:** 


*Sixty-first Year.*

VOL. LXI. MAY, 1906. No. 10

### BUFFALO MEDICAL JOURNAL

**Establishd 1845 by AUSTIN FLINT, M. D.**

LIBRARY INDEXED S. G. O.

EDITOR:

WILLIAM WARREN POTTER, M. D.

ASSISTANT EDITORS:

WILLIAM C. KRAUSS, M. D. NELSON W. WILSON, M. D.

ASSOCIATE EDITORS:
JAMES WRIGHT PUTNAM, M. D. ERNEST WENDE, M. D.
JOHN PARMENTER, M. D. JOHN A. MILLER, PH. D.
HARVEY R. GAYLORD, M. D. MAUD J. FRYE, M. D.
JOHN A. RAFTER, M. D.

